# The ability of human nuclear DNA to cause false positive low-abundance heteroplasmy calls varies across the mitochondrial genome

**DOI:** 10.1186/s12864-016-3375-x

**Published:** 2016-12-12

**Authors:** Levent Albayrak, Kamil Khanipov, Maria Pimenova, George Golovko, Mark Rojas, Ioannis Pavlidis, Sergei Chumakov, Gerardo Aguilar, Arturo Chávez, William R. Widger, Yuriy Fofanov

**Affiliations:** 1 Department of Pharmacology and Toxicology, University of Texas Medical Branch, 301 University Boulevard, Galveston, TX 77555-0144 USA; 2 Sealy Center for Structural Biology and Molecular Biophysics, University of Texas Medical Branch, Galveston, TX USA; 3 Department of Computer Science, University of Houston, Houston, TX USA; 4 Department of Physics, University of Guadalajara, Guadalajara, Jalisco Mexico; 5 Department of Biology and Biochemistry, University of Houston, Houston, TX USA

**Keywords:** Mitochondria, Heteroplasmy, Minor allele, Low-abundance mutation, NUMT, High throughput sequencing, Rare variant

## Abstract

**Background:**

Low-abundance mutations in mitochondrial populations (mutations with minor allele frequency ≤ 1%), are associated with cancer, aging, and neurodegenerative disorders. While recent progress in high-throughput sequencing technology has significantly improved the heteroplasmy identification process, the ability of this technology to detect low-abundance mutations can be affected by the presence of similar sequences originating from nuclear DNA (nDNA). To determine to what extent nDNA can cause false positive low-abundance heteroplasmy calls, we have identified mitochondrial locations of all subsequences that are common or similar (one mismatch allowed) between nDNA and mitochondrial DNA (mtDNA).

**Results:**

Performed analysis revealed up to a 25-fold variation in the lengths of longest common and longest similar (one mismatch allowed) subsequences across the mitochondrial genome. The size of the longest subsequences shared between nDNA and mtDNA in several regions of the mitochondrial genome were found to be as low as 11 bases, which not only allows using these regions to design new, very specific PCR primers, but also supports the hypothesis of the non-random introduction of mtDNA into the human nuclear DNA.

**Conclusion:**

Analysis of the mitochondrial locations of the subsequences shared between nDNA and mtDNA suggested that even very short (36 bases) single-end sequencing reads can be used to identify low-abundance variation in 20.4% of the mitochondrial genome. For longer (76 and 150 bases) reads, the proportion of the mitochondrial genome where nDNA presence will not interfere found to be 44.5 and 67.9%, when low-abundance mutations at 100% of locations can be identified using 417 bases long single reads. This observation suggests that the analysis of low-abundance variations in mitochondria population can be extended to a variety of large data collections such as NCBI Sequence Read Archive, European Nucleotide Archive, The Cancer Genome Atlas, and International Cancer Genome Consortium.

**Electronic supplementary material:**

The online version of this article (doi:10.1186/s12864-016-3375-x) contains supplementary material, which is available to authorized users.

## Background

A mitochondrion has double-stranded circular DNA, which encodes 37 genes essential for normal cell functions such as cellular energy metabolism, free radical generation, and apoptosis [1–3]. Due to its high mutation rate and only rudimentary DNA repair capabilities [4], mtDNA sequences vary among individuals, organs, and tissues [5, 6]. Furthermore, *de-novo* mtDNA mutations can accumulate over the lifetime of the individual and result in progressive deterioration of mitochondrial function [7–11]. Given that there are 2–10 copies of mtDNA per mitochondrion and up to 1000 mitochondria per cell [12], mutations in mtDNA are generally heteroplasmic, with copies of both wild-type and mutant mtDNA in each cell [13]. Low-level heteroplasmy, mitochondrial DNA mutations with minor allele frequency ≤ 1%, is associated with aging [14], cancer [15], and neurodegenerative disorders such as Alzheimer’s [16] and Parkinson’s disease [17].

Most of the techniques traditionally used to detect heteroplasmy such as Sanger capillary sequencing [18], high-performance liquid chromatography [19], SNaPshot [20], high-resolution melt profiling [21], temporal temperature gradient gel electrophoresis [22], Invader assay [23], and surveyor nuclease digestion [24] require the candidate positions to be pre-defined and do not allow determination of *de-novo* heteroplasmic locations. High Throughput Sequencing (HTS) technology allows detection of heteroplasmy across multiple locations in the mitochondrial genome simultaneously, making it the technology of choice in recent studies [13, 25–27].

However, the ability of this technology to detect heteroplasmy, especially low-abundance mutations, has its limitations. While some studies suggest that false positive rare variants can be artifacts of the sequencing technology [28] and mapping algorithms (software) [29–32], many publications have also focused on the interference of nuclear sequences of mitochondrial origin (NUMTs) on the detection of rare variants [33–35]. These studies generally consider variants with abundance below 2% potentially false positive and exclude them. The landmark work by Li et al. [28] for example, used a large number of already identified NUMTs to estimate the accuracy of low-level heteroplasmy calls and distinguish them from sequencing errors. This approach, however, relies on the reference database of NUMTs used in the analysis.

It is important to emphasize that while using only NUMTs to identify possible locations in the mitochondrial genome where nDNA can cause false positive heteroplasmy makes the computational task relatively easy, the search for NUMTs in human nuclear genomes is not yet over. Long and highly similar sequences shared between nuclear and mitochondrial DNA, also called *high fidelity* NUMTs are well described [36]. The search for new NUMTs focused on shorter and less similar subsequences continues [37, 38]. The results (potential new NUMTs) however, vary depending on the sequence similarity threshold, alignment length, and types of search algorithms used in the analysis [38].

To date, the use of paired end sequencing reads is believed to be the best way to avoid nDNA interference by making sure that both reads are mapped to the mitochondrial genome with appropriate distance between them. This assumption, however, does not take into consideration that at least 18 known NUMTs are longer that 5000 bases (out of which four are longer than 10,000 bases with the longest known to date is of the size of 14,904 bases) [39]. These NUMTs are able to produce reads pairs that may mistakenly be attributed as originating from mtDNA.

An alternative approach to minimize the effects of unknown (unidentified) NUMTs is including a “nuclear DNA exclusion” step into the heteroplasmy detection workflow. The basic idea of this method is to map all sequencing reads to the nDNA and completely exclude them from the analysis [7, 34, 35]. This approach is computationally expensive: sequencing reads from each experiment have to be mapped to approximately three gigabases long human nuclear genome. Additionally, the outcome of this approach will be significantly affected by the presence of short (starting from 11 bases) and very similar regions shared between mtDNA and nDNA sequences including, but not limited to known NUMTs. The longest shared (no mismatches) subsequence between nDNA and mtDNA is 279 bases (starting at position 4457 in the mitochondrial genome (NC_012920.1) and at position 629,627 in chromosome 1). Allowing just one mismatch, the same region of chromosome 1 extends to 417 bases (starting from positions 629,489 in chromosome 1 and position 4319 in mitochondria). The main disadvantage of the “nuclear DNA exclusion” approach is its exclusion of sequencing reads originating from such regions from consideration. This step artificially reduces coverage of the corresponding mtDNA regions and may result in false negative (missing heteroplasmy variants) outcomes.

To eliminate challenges of both approaches (using only known NUMTs and nuclear DNA exclusion) we propose to pre-compute the locations of all subsequences in the mitochondrial genome shared perfectly (no mismatches) and approximately (one mismatch allowed) between nDNA and mtDNA. This information allows to (a) avoid the nuclear DNA exclusion step so the reads only need to be mapped to the much smaller mitochondrial genome; (b) exclude ambiguous (mapped simultaneously to nuclear and mitochondrial DNA) reads; and (c) eliminate discrepancies due to the incompleteness and subjectivity in the choice of NUMTs data used in the analysis.

## Results and discussion

### Interference maps

**Fig. 1:**
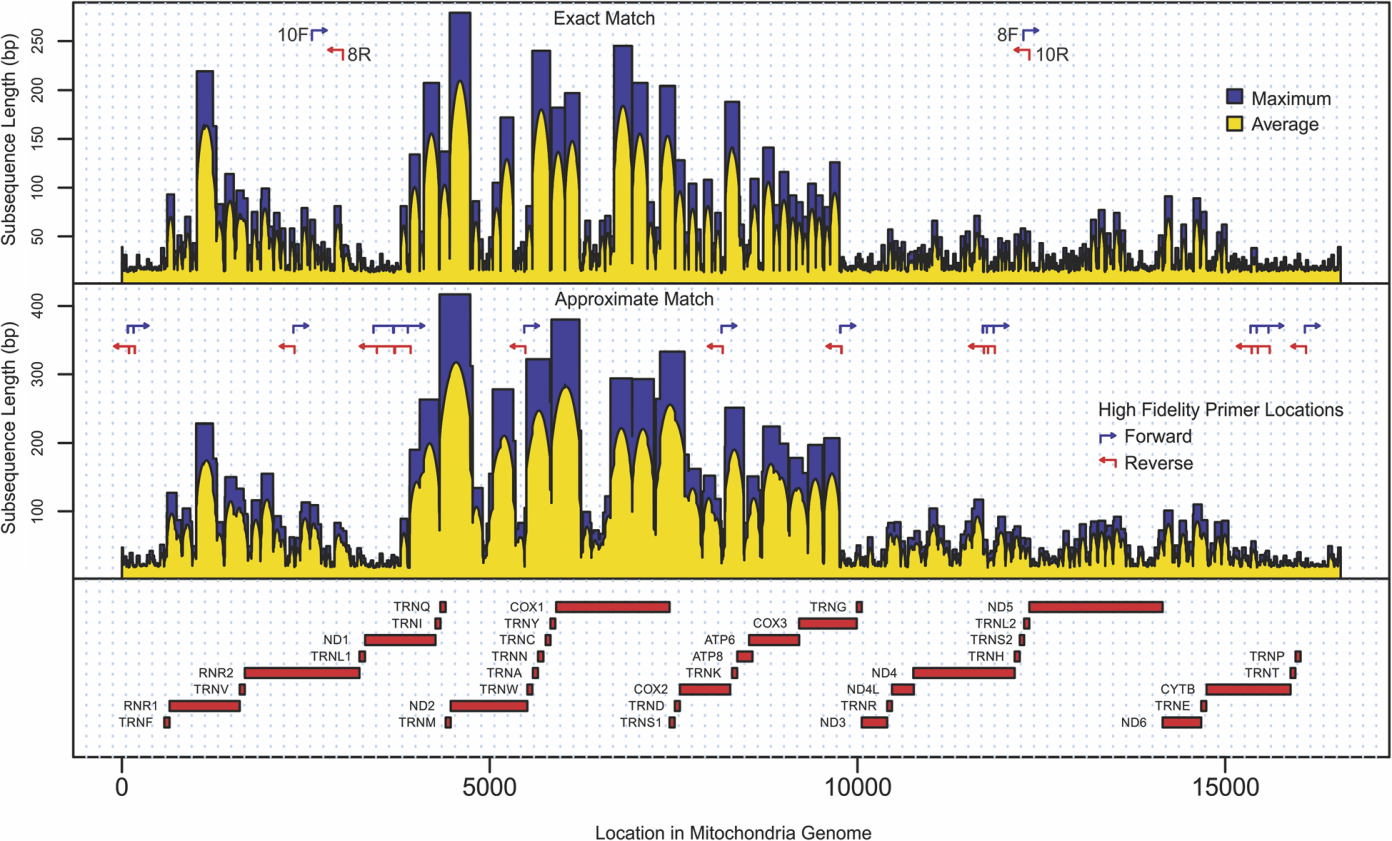
The nucleotide-by-nucleotide perfect and approximate match nDNA interference maps for the mitochondrial genome. 8F, 8R, 10F, and 10R are locations of long-range PCR primers used in Li et al. study [27]. “Maximum” (colored in *blue*) represents the length of the longest identified LCS (for exact match map) or LSS (for approximate match map) that covers an individual nucleotide position in mtDNA. “Average” (*yellow*) represents the average length of the identified LCS or LSS that covers an individual nucleotide position in mtDNA

Exhaustive comparisons of all DNA subsequences present in the human nuclear and reference mitochondrial genomes resulted in the creation of two *nuclear interference maps* for the mitochondrial genome: the *exact match map* and the *approximate match map* (Fig. 1, and Additional file 1). The *exact match map* assigns to each position in the mitochondrial genome the length of the *longest common subsequence* (LCS): the longest subsequence, which includes given position in the mitochondrial genome and present simultaneously (without mismatches) in both nuclear and mitochondrial genomes. The *approximate match map* assigns to each position in the mitochondrial genome the length of the *longest similar subsequence* (LSS): the longest subsequence, which includes given position in the mitochondrial genome and present simultaneously (with up to one mismatch difference) in both nuclear and mitochondrial genomes.

As expected, due to the presence of NUMTs and other regions containing subsequences similar to the mitochondrial genome, some common/similar subsequences between mitochondrial and nuclear DNA appear to be quite long: longest LCS and LSS among all locations in the mitochondrial genome are 279 and 417 bases in length, respectively. Performed calculations (Table 1) revealed the presence of long LCS and LSS in highly studied genes such as *ND2* (NADH dehydrogenase 2), *COX1* (cytochrome c oxidase I), *MT-COX2* (cytochrome c oxidase II), *ATP6* (ATP synthase 6), as well as *ATP8* (ATP synthase protein 8). This observation suggested that in order to avoid nDNA interference in every location in these genes, single end reads used in sequencing experiment have to be longer that 417 bases: the maximal length of LSS. In contrast, longest and average LCS and LSS in *CYTB* (cytochrome b)*, ND3* (NADH dehydrogenase 3)*, and ND4L* (NADH-ubiquinone oxidoreductase chain 4 L) genes appeared much shorter, all nDNA interference in these genes can be avoided by using single end reads as short as 100 bases. In fact, the frequency distribution of the LSS across the mitochondrial genome suggests that for approximately 20.4% using only 36 bases long single end reads will be enough to avoid the nDNA interference (44.5 and 67.9% for 76 and 150 bases long reads, respectively). This opens the opportunity to extend analysis of low-abundance variation in mitochondrial population to large data collections such as NCBI Sequence Read Archive [40], European Nucleotide Archive [41], The Cancer Genome Atlas [42], and International Cancer Genome Consortium [43] which were by no means designed to explore the low-level heteroplasmy in mitochondrial genome.

**Table 1:** Longest common and longest similar subsequences in mitochondrial genes

Gene Name	Length	Start Position	End Position	Maximum Length of Longest Common Subsequences	Average Length of Longest Common Subsequences	Maximum Length of Longest Similar Subsequences	Average Length of Longest Similar Subsequences
*TRNF*	71	577	647	93	55.2	127	85.2
*RNR1*	954	648	1601	219	108.3	228	139.9
*TRNV*	69	1602	1670	97	95.0	133	128.8
*RNR2*	1559	1671	3229	99	51.3	155	81.1
*TRNL1*	75	3230	3304	42	28.6	46	37.3
*ND1*	956	3307	4262	207	74.0	263	108.6
*TRNI*	69	4263	4331	207	174.8	417	285.9
*TRNQ*	72	4400	4329	137	137.0	417	417.0
*TRNM*	68	4402	4469	279	162.7	417	417.0
*ND2*	1042	4470	5511	279	131.9	417	242.3
*TRNW*	68	5512	5579	81	81.0	322	322.0
*TRNA*	69	5655	5587	240	240.0	322	322.0
*TRNN*	73	5729	5657	240	240.0	322	322.0
*TRNC*	66	5826	5761	240	219.8	322	316.3
*TRNY*	66	5891	5826	182	144.7	380	351.6
*COX1*	1542	5904	7445	245	145.7	380	255.8
*TRNS1*	69	7514	7446	204	204.0	333	333.0
*TRND*	68	7518	7585	204	129.8	333	333.0
*COX2*	684	7586	8269	188	86.4	333	169.4
*TRNK*	70	8295	8364	188	188.0	251	251.0
*ATP8*	207	8366	8572	188	76.2	251	177.3
*ATP6*	681	8527	9207	141	97.4	224	183.6
*COX3*	784	9207	9990	126	71.4	207	140.4
*TRNG*	68	9991	10058	32	29.4	50	45.0
*ND3*	346	10059	10404	41	24.4	76	36.6
*TRNR*	65	10405	10469	57	49.4	83	81.6
*ND4L*	297	10470	10766	57	33.6	84	63.6
*ND4*	1378	10760	12137	71	37.1	117	70.6
*TRNH*	69	12138	12206	52	44.9	78	78.0
*TRNS2*	59	12207	12265	58	50.3	78	62.3
*TRNL2*	71	12266	12336	58	51.8	61	59.6
*ND5*	1812	12337	14148	77	34.1	92	53.0
*ND6*	525	14673	14149	91	52.4	110	78.5
*TRNE*	69	14742	14674	75	75.0	110	89.0
*CYTB*	1141	14747	15887	62	27.4	87	43.3
*TRNT*	66	15888	15953	26	20.0	30	24.0
*TRNP*	68	15956	16023	32	26.6	40	36.1

**Fig. 2:**
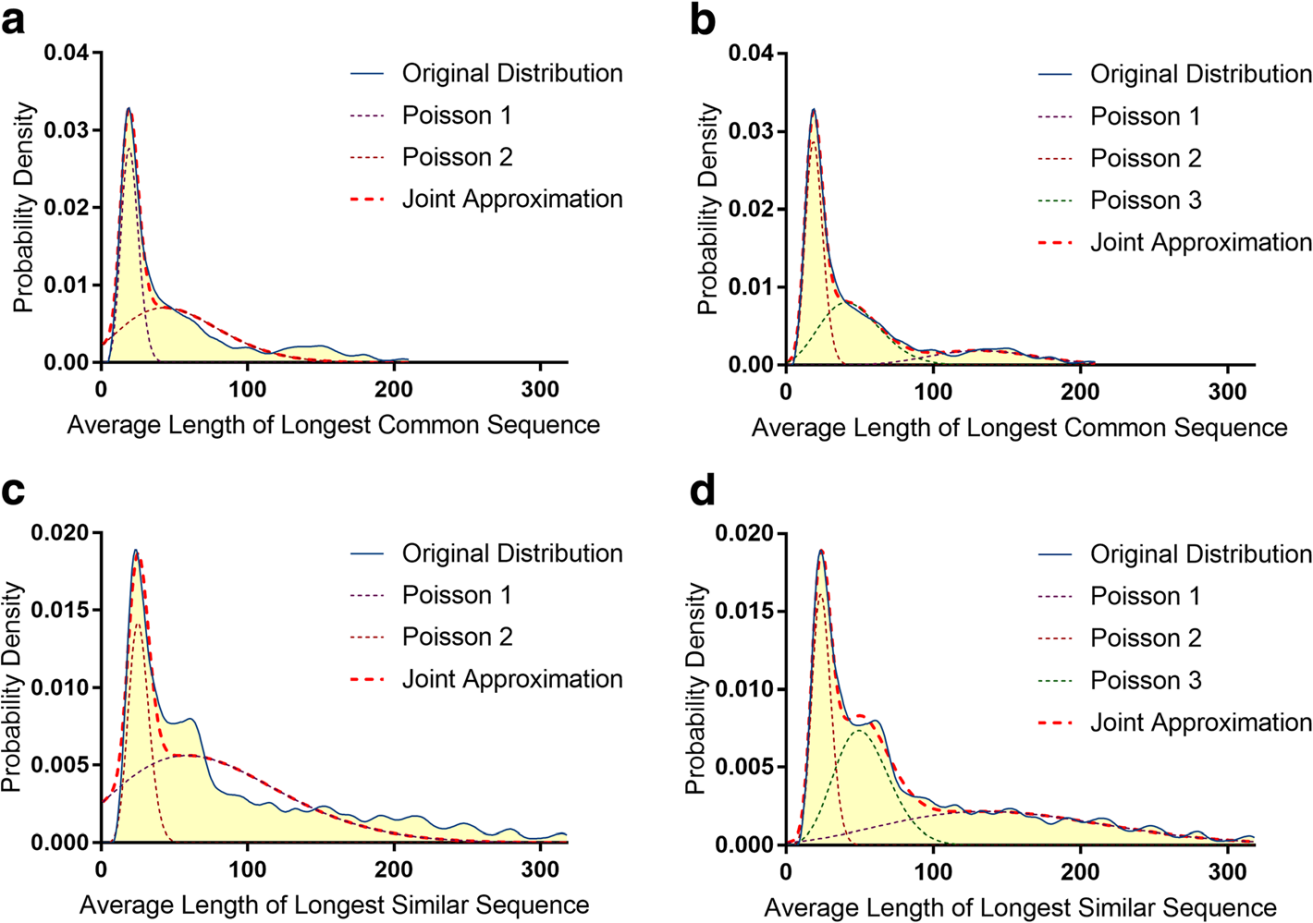
Poisson distributions. Approximation of average length of LCS and LSS covering each position in the mitochondrial genome by two (**a**, **c**) and three (**b**, **d**) mixed Poisson distributions. The *red dashed line* represents the joint approximation of the mixed distributions

Surprisingly, we observed more than a 25-fold difference in the length of both LCS and LSS across different locations in the mitochondrial genome. The shortest LCS was only 11 bases long and appeared in two locations in the *ND5* gene. The shortest LSS was 15 bases long and appeared in two locations of the hypervariable D-loop region. As one can see in Fig. 1, the locations of the LCS and LSS do not appear to be random and express a bias to particular regions of the mitochondrial genome. For example, the region located between positions 4000 and 10,000 in the mitochondrial genome contains the vast majority of long LCS and LSS while the region between positions 10,000 and 16,000 contains a high concentration of short LCS and LSS. One possible explanation of these results can be that the introduction of mitochondrial sequences into the nuclear genome has a bias against certain locations in mtDNA (such as the 10–16 k region). Another possibility is that some mtDNA sequences have been introduced into nuclear DNA over earlier periods of evolution and have accumulated more mutations than other sequences that have been more recently transferred from mtDNA to nDNA. The length distributions of LCS and LSS that almost perfectly fit into a mixture of two and three Poisson distributions (Fig. 2a-d) also suggested the involvement of several different mechanisms behind the origin/evolution of shared mtDNA and nDNA subsequences. These observations are also in agreement with recent work by Tsuji et al. [44] reporting the underrepresentation of D-Loop sequences in nDNA. Performed analysis, however, pointed to several additional locations in the mitochondrial genome (including regions within the *RNR1*, *TRNL1*, and *ND1* genes) exhibiting properties similar to D-loop sequences (i.e. being distant from nDNA) suggesting that these regions could be mutated and/or underrepresented in nDNA (Fig. 1 and Table 1).

### Long range PCR primers

**Fig. 3:**
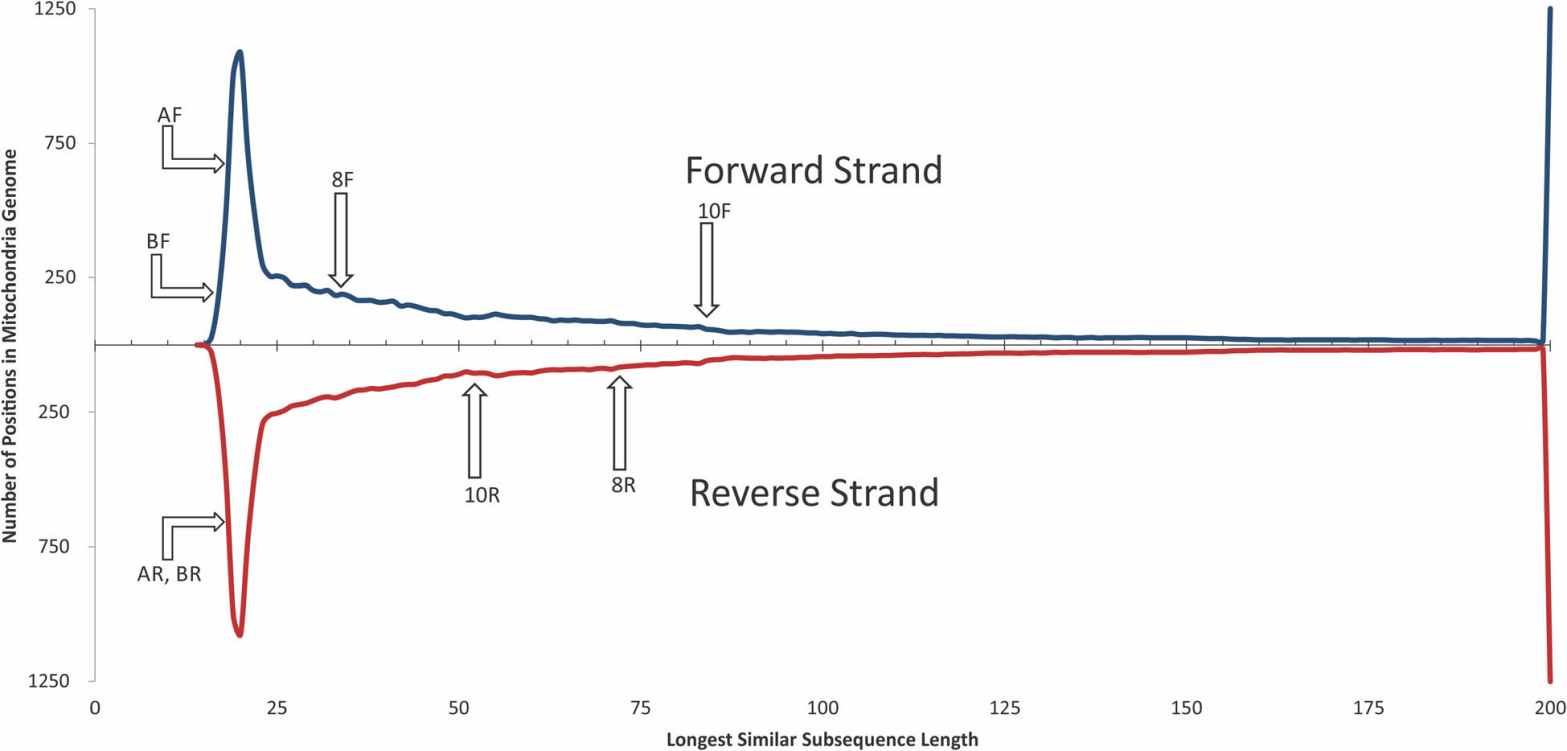
The length distribution of LSS in the mitochondrial genome. 8F, 8R, 10F, and 10R are locations of forward (F) and reverse (R) primers used for long-range mtDNA amplification by Li et al [27]. AF, AR, BF, and BR are the primers designed to be located in the regions with the lowest similarity to the nuclear genome

The noticeable presence of short subsequences unique to mtDNA contradicts the common opinion that due to the NUMTs, primers designed for mtDNA amplification will always amplify (perhaps linearly) some amount of nDNA. Although this is true for the primers originally designed by Meyer et al. for use in long-range amplification [45] and employed in the Li et al. studies [27, 28, 46] (the locations of primer pairs for 8 and 10 kb overlapping mtDNA regions can be found in Fig. 1), performed analysis suggests that each of these aforementioned primers is not unique to mtDNA and has many similar sequences in nDNA (Table 2, Fig. 3). Furthermore, the analysis of the frequency distribution of the length of LCS and LSS revealed that 39 and 72% of locations, respectively, in the mitochondrial genome are more specific than the locations chosen for these primers. *In-silico* PCR, using MFEprimer-3.0 [47] against the human genome, shows that these primers can produce 24 amplicons from the human nuclear genome (see Additional file 2 for more information).

**Table 2:** Characteristics of four long-range mtDNA PCR primers used by Li et al [27] and four proposed high fidelity long-range primers

Primers set	Primer	Sequence	Number of similar sequences in nDNA	Primer length/LSS starting from primers position	Number of positions in mtDNA with less value of LSS	Percent of positions in mtDNA with less value of LSS
Long-range mtDNA PCR primers used by *Li et al* [25]	10 F (forward_10kb)	CCGTGCAAAGGTAGCATAATC	8	21/84	11,965	72.21%
	10R (reverse_10kb)	TTACTTTTATTTGGAGTTGCACCA	16	24/52	9,125	55.07%
	8 F (forward_8kb)	GGCTTTCTCAACTTTTAAAGGATA	10	24/34	6,504	39.25%
	8R (reverse_8kb)	TGTCCTGATCCAACATCGAG	14	20/72	11,089	66.93%
Proposed long-range mtDNA PCR primers	AF (forward_10kb)	TACTACCAGACAACCTTAGCCA	0	22/18	218	1.32%
	AR (reverse_10kb)	GAGTCATAAGTGGAGTCCGTAA	0	22/18	217	1.31%
	BF (forward_8kb)	CACCATTTCCGACGGCATCTAC	0	22/16	1	0.01%
	BR (reverse_8kb)	TGCGCCAGGTTTCAATTTCTAT	0	22/18	217	1.31%

The best locations for highly specific PCR primers would be positions where the lengths of the LSS are at their minima. Several such locations, which are highly specific for mtDNA, are shown in Fig. 1. As an example, we tested four of such location and designed new long-range PCR primers (Table 2, Fig. 3) to amplify this region. *In-silico* PCR using these primers and a human genome target shows no viable nDNA amplicons (see Additional file 2 for more information). An in-vivo validation using Illumina MiSeq at 151 cycles followed by mapping to the mitochondrial genome (NC_012920.1) and the human genome (GRCh38) shows that the proposed primers allowed a 10% reduction in the proportion of reads mapped to the nuclear genome at 95% alignment identity (see Methods section for more details). It is important to emphasize, however, that proposed primers are only an example of how the fidelity of long-range PCR can be improved. Figure 1 shows several (out of many) possible locations for long-range PCR primers. Testing all the mitochondria-specific locations could lead to further improvement of the mtDNA enrichment protocol but it is beyond the scope of this manuscript.

It is important to emphasize the limitations of the presented interference maps. The latest human genome build (GRCh38) used in the analysis is missing some sequences from highly repetitious regions, especially centromeres. Additionally, personal genomic variations (single nucleotide polymorphisms and structural variations) and possible variations in NUMTs between individuals were not included in our computations. More robust interference maps can be built using a higher number of mismatches. For these reasons, the presented interference maps only allow the identification of the minimum required sequencing read length in mitochondrial regions of interest and establishes a confidence level in the analysis results.

## Conclusion

Due to high throughput and rapidly decreasing cost, HTS offers new opportunities to identify mitochondrial heteroplasmy including low-level heteroplasmy. The presence of a significant amount of nDNA, however, can introduce bias and cause false positive low-level heteroplasmy calls. This is especially important for in-solution capturing techniques, which have become more popular for mitochondrial heteroplasmy analysis over the last few years [48–51].

To avoid interference from nDNA at each given location, the length of the LSS at that location must be longer than the length of the sequencing reads. Proposed maps allow to predict the level of possible nDNA interference for each location in the mitochondrial genome, improve the fidelity of rare variant identification, and guide the choice of the sequencing read length. Performed analysis suggests that heteroplasmy (including low-level heteroplasmy) can be identified using short (36, 76, and 150 bases long) single end read for the significant part of the mitochondrial genome, which opens an opportunity to employ the data from a variety of existing collections such as the Sequence Read Archive and The Cancer Genome Atlas into the analysis.

Identified locations of short LCS and LSS in the mitochondrial genome can also be used to improve the quality of long-range PCR primers and probes for in-solution mtDNA capture by eliminating binding to the nuclear genome. The locations and length distributions of LCS and LSS support the hypothesis of non-random mtDNA introduction into human nuclear DNA and suggest that several regions of the mitochondrial genome (in addition to the previously reported D-loop) may be underrepresented or mutated after being introduced into nDNA.

## Methods

### Nuclear, mitochondrial, and NUMTs data

Presented analyses were performed using the human reference genome (GRCh38). In addition to the sequences of the 24 chromosomes, this build contains 430 unlocalized scaffolds (genomic subsequences with an identified chromosome of origin but an unknown location or orientation) totaling 120,999,704 bases in length. It is important to emphasize that the widely accepted mitochondrial reference genome (NC_012920.1) used in the presented analysis contains an unknown nucleotide “N” (position 3106) which was artificially introduced during the revision of the Cambridge reference sequence of human mitochondrial DNA [2] to maintain position numbering consistent with previous literature [2]. During mapping and analysis, this nucleotide was removed from the reference sequence and restored after mapping. The *longest common* and *longest similar* sequence lengths for this position were assigned to be equal to the corresponding values for the previous (3105) position. The NUMTs database uploaded by Simone et al. [38] in an unpublished study, “Revised RHNumtS compilation,” was downloaded from the NCBI Nucleotide database [39] in June 2015. The list of accession numbers for the NUMTs is available in the Additional file 2 document.

### HTS data and analysis

In order to find the LCS and LSS between human nuclear DNA and mitochondrial DNA, all human reference sequences were disassembled into overlapping subsequences of 450 bases so that each location in the human genome was associated with two (original and reverse complement) disassembled subsequences. The initial choice of the length of sequences used in the analysis (450 bases) has been made under the assumption that the maximum length of LSS will be lower than this value (which appeared to be 417). Otherwise, this value would be increased and calculations would be repeated. Human-derived subsequences were de-replicated (duplications excluded) for future calculations. The nDNA subsequences were placed in a search efficient data structure (combination of a sorted array and suffix tree) allowing to perform an exhaustive search with mismatches (insertions, deletions, and substitutions) for each subsequence and all of its prefixes. Each prefix of every 450 base-long subsequence derived from the mitochondrial genome (including complementary sequence) was searched against this data structure with perfect matching to identify the LCS and with one permitted mismatch to identify the LSS starting from each position. Software specifically developed for this purpose was used in the computations performed on the Lonestar high-performance computing cluster at the Texas Advanced Computing Center (The University of Texas at Austin).

The interference maps were created by identifying each of the *longest common* and *longest similar* subsequences that included the given position and calculated the maximum and average length of such subsequences (Fig. 1, and Additional file 1).

### Poisson fitting

A Gaussian convolution kernel filter (*μ* = 21 *and σ = 3*) was used to reduce the effects of noise in the probability density functions of average LCS and LSS lengths (Fig. 2). The statistical models of the distribution were defined as mixtures of two and three linearly transformed Poisson distributions where the probability density of LSS and LCS of the length (*l*) was defined as:

$$ G\left(l,\alpha, \beta, \lambda, k\right)={\displaystyle \sum_{i=1}^k}{\alpha}_i{\beta}_iP\left({\lambda}_i,{\beta}_il\right) $$

where

- *α*_1_,.., *α*_2_ *= proportions of Poisson distributions in the mixture* ${\displaystyle \sum_{i=1}^k}{\alpha}_i=1;$
- *β*_1_,.., *β*_2_ *= transformation coefficients of Poisson distribution;*
- *λ*_1_,.., *λ*_2_ *= parameters of Poisson distributions;*
- *k = number of Poisson distributions in the mixture.*

The fitness function was defined as the sum of squares of deviations of the model from the observed probability density and was optimized using all 3 k − 1 parameters of the mixture of transformed Poisson distributions (*N* = *number of observations*, *F*(*l*_*i*_) = *probability density at length* ' *i* '):

$$ \underset{\alpha_1,..,{\alpha}_k}{ \min}\kern0.5em \underset{\beta_1,..,{\beta}_k}{\  \min}\kern0.5em \underset{\lambda_1,..,{\lambda}_k}{\  \min }{\displaystyle \sum_{i=1}^N}{\left(F\left({l}_i\right)-G\left({l}_i,\alpha, \beta, \lambda, k\right)\right)}^2 $$

The error and parameter values for when the fitness function reaches its minimum can be found in Table 3.

**Table 3:** Error and parameter values identified for average length of LCS and LSS distributions using two and three Poisson mixtures

	LCS		LSS	
	Mixture of 2 Poisson Distributions *(k = 2)*	Mixture of 3 Poisson Distributions *(k = 3)*	Mixture of 2 Poisson Distributions *(k = 2)*	Mixture of 3 Poisson Distributions *(k = 3)*
*α*_1_	0.4071	0.1709	0.7556	0.3993
*α*_2_	0.5929	0.4195	0.2444	0.2574
*α*_3_		0.4097		0.3433
*β*_1_	0.5729	0.1030	0.0263	0.0277
*β*_2_	0.0472	0.5691	0.5456	0.5980
*β*_3_		0.1090		0.1511
*λ*_1_	11.3771	14.0520	2.0610	4.2692
*λ*_2_	2.5450	11.0717	14.1339	14.5746
*λ*_3_		4.96376		7.9806
*error*	2.8675E-04	3.3724E-05	4.9989E-04	3.6505E-05

### PCR primer design and validation

Two pairs of primers (pair A and pair B) amplifying overlapping 10 and 8 kb regions, respectively, of the mitochondrial genome, were selected from regions with the lowest similarity to the nuclear genome using the proposed interference maps. Primer validation was performed using *in-silico* PCR using MFEprimer-3.0 [46]. The same software has been used to evaluate the primers employed by *Li* et al. [25]. Both specific and non-specific amplification was predicted for all primers using the human reference genome (GRCh38) (see Additional file 2 for more information). An in-vivo validation of the proposed primers was performed using human DNA from a de-identified archived specimen. DNA was isolated using DNeasy Blood and Tissue kit (Qiagen, CA, USA). The isolated DNA was amplified using the LongAmp Taq PCR Kit (New England Bio Labs, MA, USA) under three conditions, varying the annealing temperature. More details including the PCR amplification conditions can be found in the Additional file 2.

Sequencing has been performed using Illumina MiSeq instrument at 151 cycles. The read filtration step included trimming reads containing: (a) nucleotides below the quality threshold of 0.05 (using modified Richard Mott algorithm); (b) two or more unknown nucleotides; and (c) Nextera tagmentation library adapters. Reads from each dataset were mapped to the mitochondrial genome (NC_012920.1) and the human genome (GRCh38, chromosomes only) using the CLC Genomics Workbench 9.0.1 “*Map Reads to Contigs”* analysis tool. The proposed primers showed approximately a 10% reduction in mapping to the human genome at 95% alignment identity (see Additional file 2 for more information).

## Additional files

Additional file 1: Exact and approximate match nuclear DNA interference maps for the mitochondrial genome. (XLSX 1715 kb)

Additional file 2: Supplementary Materials Document. (DOCX 454 kb)

## Abbreviations

HTS: High throughput sequencing
LCS: Longest common subsequence
LSS: Longest similar subsequence
mtDNA: Mitochondrial DNA
nDNA: Nuclear DNA
NUMT: Nuclear sequence of mitochondrial origin
