# Supplementary material for: The ability of human nuclear DNA to cause false positive low-abundance heteroplasmy calls varies across the mitochondrial genome
Source: BMC Genomics. 2016 Dec 12;17:1017. doi: 10.1186/s12864-016-3375-x (PMC5153897; doi:10.1186/s12864-016-3375-x)
Supplement: Supplementary file 2 — Supplementary Materials Document. (DOCX 454 kb) [file 12864_2016_3375_MOESM2_ESM.docx]

**Additional file 2**

**The Ability of Human Nuclear DNA to Cause False Positive Low-Abundance Heteroplasmy Calls Varies Across the Mitochondrial Genome**

Levent Albayrak^a,b,c^, Kamil Khanipov^a,b,c^, Maria Pimenova^a,b^, George Golovko^a,b^, Mark Rojas^a,b^, Ioannis Pavlidis^c^, Sergei Chumakov^d^, Gerardo Aguilar^d^, Arturo Chávez^d^, William R. Widger^e^, Yuriy Fofanov^a,b^

^a^ Dept. of Pharmacology and Toxicology, University of Texas Medical Branch, Galveston, Texas, USA

^b^ Sealy Center for Structural Biology and Molecular Biophysics, University of Texas Medical Branch, Galveston, Texas, USA

^c^ Dept. of Computer Science, University of Houston, Houston, Texas, USA

^d^ Dept. of Physics, University of Guadalajara, Guadalajara, Jalisco, Mexico

^e^ Dept. of Biology and Biochemistry, University of Houston, Houston, Texas, USA

Levent Albayrak (LA) [lealbayr@utmb.edu](mailto:lealbayr@utmb.edu), Kamil Khanipov (KK) [kakhanip@utmb.edu](mailto:kakhanip@utmb.edu), Maria Pimenova (MP) [mapimeno@utmb.edu](mailto:mapimeno@utmb.edu), Georgiy Golovko (GG) [gegolovk@utmb.edu](mailto:gegolovk@utmb.edu), Mark Rojas (MR) [mmrojas@utmb.edu](mailto:mmrojas@utmb.edu), Ioannis Pavlidis (IP) [ipavlidis@uh.edu](mailto:ipavlidis@uh.edu), Sergei Chumakov (SC) [sergei@cencar.udg.mx](mailto:sergei@cencar.udg.mx), Gerardo Aguilar (GA) [gerardo.solano@mxro.io](mailto:gerardo.solano@mxro.io), Arturo Chávez (AC) [divcb@cucei.udg.mx](mailto:divcb@cucei.udg.mx), William R. Widger (WW) [widger@uh.edu](mailto:widger@uh.edu)

Corresponding Author:

Yuriy Fofanov (YF) [yufofano@UTMB.EDU](mailto:yufofano@UTMB.EDU)

Department of Pharmacology & Toxicology,

301 University Boulevard, Galveston, Texas, 77555-0144

**Section A. NCBI Accession Numbers of the NUMTs Used in Analysis.**

**Table S1. NCBI Nucleotide Database Accession Numbers [1, 2]**

| FN674062.1 | FN674037.1 | FN673990.1 | FN673934.1 | FN673891.1 | FN673847.1 | HE613814.1 |
| --- | --- | --- | --- | --- | --- | --- |
| FN673953.1 | FN674036.1 | FN673989.1 | FN673933.1 | FN673890.1 | FN673846.1 | HE613813.1 |
| FN673952.1 | FN674035.1 | FN673987.1 | FN673932.1 | FN673889.1 | FN673845.1 | HE613812.1 |
| FN674048.1 | FN674034.1 | FN673986.1 | FN673931.1 | FN673888.1 | FN673844.1 | HE613811.1 |
| FN674038.1 | FN674033.1 | FN673984.1 | FN673930.1 | FN673887.1 | FN673843.1 | HE613810.1 |
| FN674032.1 | FN674030.1 | FN673983.1 | FN673929.1 | FN673886.1 | FN673842.1 | HE613809.1 |
| FN674031.1 | FN674028.1 | FN673982.1 | FN673928.1 | FN673885.1 | FN673841.1 | HE613808.1 |
| FN674029.1 | FN674027.1 | FN673981.1 | FN673927.1 | FN673884.1 | FN673840.1 | HE613807.1 |
| FN674013.1 | FN674026.1 | FN673980.1 | FN673926.1 | FN673883.1 | FN673839.1 | HE613806.1 |
| FN673988.1 | FN674025.1 | FN673979.1 | FN673925.1 | FN673882.1 | HE613848.1 | HE613805.1 |
| FN673985.1 | FN674024.1 | FN673977.1 | FN673924.1 | FN673881.1 | HE613847.1 | HE613804.1 |
| FN673978.1 | FN674023.1 | FN673975.1 | FN673923.1 | FN673880.1 | HE613846.1 | HE613803.1 |
| FN673976.1 | FN674022.1 | FN673974.1 | FN673922.1 | FN673879.1 | HE613845.1 | HE591379.1 |
| FN673964.1 | FN674021.1 | FN673973.1 | FN673921.1 | FN673878.1 | HE613844.1 | HE591378.1 |
| FN673957.1 | FN674020.1 | FN673972.1 | FN673920.1 | FN673877.1 | HE613843.1 | HE591377.1 |
| FN673956.1 | FN674019.1 | FN673971.1 | FN673919.1 | FN673876.1 | HE613842.1 | HE591376.1 |
| FN673955.1 | FN674018.1 | FN673970.1 | FN673918.1 | FN673875.1 | HE613841.1 | HE591375.1 |
| FN673954.1 | FN674017.1 | FN673969.1 | FN673917.1 | FN673874.1 | HE613840.1 | HE591374.1 |
| FN673951.1 | FN674016.1 | FN673968.1 | FN673916.1 | FN673873.1 | HE613839.1 | HE591373.1 |
| FN673950.1 | FN674015.1 | FN673967.1 | FN673915.1 | FN673872.1 | HE613838.1 | FR850287.1 |
| HE613849.1 | FN674014.1 | FN673966.1 | FN673914.1 | FN673871.1 | HE613837.1 | FR850286.1 |
| FN674041.1 | FN674012.1 | FN673965.1 | FN673913.1 | FN673870.1 | HE613836.1 | FR850285.1 |
| FN674061.1 | FN674011.1 | FN673963.1 | FN673912.1 | FN673869.1 | HE613835.1 | FR850284.1 |
| FN674060.1 | FN674010.1 | FN673962.1 | FN673911.1 | FN673868.1 | HE613834.1 | FR850283.1 |
| FN674059.1 | FN674009.1 | FN673961.1 | FN673910.1 | FN673867.1 | HE613833.1 | FR850282.1 |
| FN674058.1 | FN674008.1 | FN673960.1 | FN673909.1 | FN673866.1 | HE613832.1 | FR850281.1 |
| FN674057.1 | FN674007.1 | FN673959.1 | FN673908.1 | FN673865.1 | HE613831.1 | FR850280.1 |
| FN674056.1 | FN674006.1 | FN673958.1 | FN673907.1 | FN673864.1 | HE613830.1 | FR850279.1 |
| FN674055.1 | FN674005.1 | FN673949.1 | FN673906.1 | FN673863.1 | HE613829.1 | FR850278.1 |
| FN674054.1 | FN674004.1 | FN673948.1 | FN673905.1 | FN673862.1 | HE613828.1 | FR850277.1 |
| FN674053.1 | FN674003.1 | FN673947.1 | FN673904.1 | FN673860.1 | HE613827.1 | FR850276.1 |
| FN674052.1 | FN674002.1 | FN673946.1 | FN673903.1 | FN673859.1 | HE613826.1 | FR850275.1 |
| FN674051.1 | FN674001.1 | FN673945.1 | FN673902.1 | FN673858.1 | HE613825.1 | FR850274.1 |
| FN674050.1 | FN674000.1 | FN673944.1 | FN673901.1 | FN673857.1 | HE613824.1 | FR850273.1 |
| FN674049.1 | FN673999.1 | FN673943.1 | FN673900.1 | FN673856.1 | HE613823.1 | FR850272.1 |
| FN674047.1 | FN673998.1 | FN673942.1 | FN673899.1 | FN673855.1 | HE613822.1 | FR850271.1 |
| FN674046.1 | FN673997.1 | FN673941.1 | FN673898.1 | FN673854.1 | HE613821.1 | FR850270.1 |
| FN674045.1 | FN673996.1 | FN673940.1 | FN673897.1 | FN673853.1 | HE613820.1 | FR850269.1 |
| FN674044.1 | FN673995.1 | FN673939.1 | FN673896.1 | FN673852.1 | HE613819.1 | FR850268.1 |
| FN674043.1 | FN673994.1 | FN673938.1 | FN673895.1 | FN673851.1 | HE613818.1 | FR850267.1 |
| FN674042.1 | FN673993.1 | FN673937.1 | FN673894.1 | FN673850.1 | HE613817.1 | FR850266.1 |
| FN674040.1 | FN673992.1 | FN673936.1 | FN673893.1 | FN673849.1 | HE613816.1 | FR850265.1 |
| FN674039.1 | FN673991.1 | FN673935.1 | FN673892.1 | FN673848.1 | HE613815.1 |  |

**Section B. Primer validation**

**Section B1. *In-silico* PCR Primer Validation**

Primer specificity was validated using *in-silico* PCR by MFEprimer-3.0 program [3]. Non-specific amplification by the mix of four primers was evaluated using human reference RNA and genome sequences (GRCh38) as the background, allowing for amplicons between 50 and 100,000 bases.

**Table S2. Sequences and locations of existing long-range PCR primers [4]**

| Primer | Starting Position in Mitochondria | Size (bp) | Nucleotide Sequence |
| --- | --- | --- | --- |
| 10F | 2,583 | 21 | CCGTGCAAAGGTAGCATAATC |
| 10R | 12,360 | 24 | TTACTTTTATTTGGAGTTGCACCA |
| 8F | 12,255 | 24 | GGCTTTCTCAACTTTTAAAGGATA |
| 8R | 3,025 | 20 | TGTCCTGATCCAACATCGAG |

**Table S3. Predicted PCR products (amplicons) for existing long range mtDNA PCR primers [4]**

| Chromosome Hit | Fp x Rp | Product Size (bp) | PPC (%) | Fp Tm (°C) | Rp Tm (°C) |
| --- | --- | --- | --- | --- | --- |
| Chromosome 5 | 10R x 8F | 82 | 100 | 57.88 | 55.95 |
| Chromosome 4 | 10R x 8F | 82 | 100 | 57.88 | 55.95 |
| Chromosome 4 | 8R x 10F | 423 | 96.55 | 57.61 | 57.96 |
| Chromosome 3 | 10F x 8R | 422 | 96.55 | 57.96 | 57.61 |
| Chromosome X | 8R x 10F | 422 | 96.55 | 57.61 | 52.37 |
| Chromosome 9 | 10F x 8R | 425 | 95.24 | 55.43 | 57.61 |
| Chromosome 4 | 8F x 10R | 82 | 84.03 | 50.37 | 57.31 |
| Chromosome 7 | 10F x 8R | 425 | 79.33 | 50.12 | 57.61 |
| Chromosome 7_KI270803v1_alt | 10F x 8R | 425 | 79.33 | 50.12 | 57.61 |
| Chromosome 21* | 10R x 8F | 81 | 66.15 | 57.88 | 46.17 |
| Chromosome X* | 10R x 8F | 82 | 59.85 | 57.88 | 37.74 |
| Chromosome 17* | 10F x 8R | 423 | 57 | 39.66 | 57.61 |
| Chromosome 20* | 8R x 10F | 417 | 50.03 | 57.61 | 37.31 |
| Chromosome 4* | 10F x 8R | 400 | 43.37 | 57.96 | 39.68 |
| Chromosome 2* | 10F x 8R | 420 | 43.33 | 34.61 | 57.61 |
| Chromosome 5* | 10R x 8F | 82 | 36.62 | 57.88 | 32.32 |
| Chromosome X* | 10F x 8R | 47582 | 35.04 | 30.88 | 39.68 |
| Chromosome 17* | 10F x 10R | 70713 | 34.78 | 39.66 | 38.18 |
| Chromosome 16* | 10R x 10F | 49340 | 34.78 | 31.67 | 39.66 |
| Chromosome 2* | 10R x 8F | 67373 | 34.68 | 37.99 | 32.32 |
| Chromosome 8* | 10F x 10F | 88728 | 32.65 | 30.88 | 30.88 |
| Chromosome 4* | 10R x 10R | 73048 | 31.39 | 57.88 | 38.18 |
| Chromosome 16* | 8R x 8R | 29188 | 30.25 | 33.62 | 33.62 |
| Chromosome 13* | 8R x 10R | 642 | 30 | 37.51 | 33.21 |
| Fp – Forward primer;  Rp – Reverse primer;  PPC (%) – Primer Pair Coverage;  Fp Tm – Forward primer melting temperature in ̊C;  Rp Tm – Reverse primer melting temperature in ̊C;  * – For this product one of the primer’s melting temperatures is below 50 ̊C, making it highly unlikely to produce an actual product. | | | | | |

**Table S4. Sequences and locations of proposed long range mtDNA PCR primers**

| Primer | Starting Position in Mitochondria | Size (bp) | Nucleotide Sequence | Tm (°C) |
| --- | --- | --- | --- | --- |
| AF | 1701 | 22 | TACTACCAGACAACCTTAGCCA | 58.82 |
| AR | 11407 | 22 | GAGTCATAAGTGGAGTCCGTAA | 57.61 |
| BF | 9764 | 22 | CACCATTTCCGACGGCATCTAC | 62.20 |
| BR | 1772 | 22 | TGCGCCAGGTTTCAATTTCTAT | 59.50 |

**Table S5. Predicted PCR products (amplicons) for proposed long range mtDNA PCR primers**

| Chromosome Hit | Fp x Rp | Product Size (bp) | PPC (%) | Fp Tm (°C) | Rp Tm (°C) |
| --- | --- | --- | --- | --- | --- |
| Chromosome 2* | AF x BR | 42591 | 30.93 | 37.43 | 32.46 |
| Fp – Forward primer;  Rp – Reverse primer;  PPC (%) – Primer Pair Coverage;  Fp Tm – Forward primer melting temperature in ̊C;  Rp Tm – Reverse primer melting temperature in ̊C;  * – For this product one of the primer’s melting temperatures is below 50 ̊C, making it highly unlikely to produce an actual product. | | | | | |

**Section B2. *In-vivo* PCR Primer Validation**

*In-vivo* validation of proposed long-range PCR primers was performed using archived human DNA. DNA was isolated using DNeasy Blood and Tissue kit (Qiagen, CA, USA). The manufacturer’s protocol for isolation was followed with the addition of two steps: (1) pre-treatment with lysozyme solution (180 µl of solution; incubation 30 min at 37 ˚C); (2) extra treatment with RNase A (4 µl of RNase A (100mg/ml), followed by vortex and incubation for 2 min at room temperature). The mtDNA was amplified by the LongAmp Taq PCR Kit (New England Bio Labs, MA, USA) using existing and newly proposed long-range PCR primers. The amplification was done under three conditions to find the optimal annealing temperature:

Condition 1. initial denaturation at 94 °C for 30 seconds, then 30 cycles at 94°C for 15 seconds, 52 °C for 15 seconds and 65 °C for 8 min 22 sec, followed by a 10-minute final extension at 65°C;

Condition 2. initial denaturation at 94 °C for 30 seconds, then 30 cycles at 94°C for 30 seconds, 52 °C for 30 seconds and 65 °C for   9 min, followed by a 10-minute final extension at 65°C;

Condition 3. initial denaturation at 94 °C for 30 seconds, then 30 cycles at 94°C for 30 seconds, 54 °C for 30 seconds and 65 °C for 9 min, followed by a 10-minute final extension at 65°C.

The PCR clean-up was performed using Agencourt AMPure XP (Beckman Coulter, Irving, TX) at a 1.5X concentration. Post clean-up the amplification products were visualized on a 1% agarose gel with a HyperLadder 1 kb (Bioline, MA, USA) (Supplementary Figure 1). The amplicons from conditions two and three were then prepared into sequencing libraries using the Nextera DNA Library Preparation kit and sequenced on an Illumina Miseq at 151 cycles. Reads filtration was performed by trimming reads containing: (a) nucleotides below the quality threshold of 0.05 (using modified Richard Mott algorithm); (b) two or more unknown nucleotides; and (c) Nextera tagmentation library adapters. Reads from each dataset were mapped to the mitochondrial genome (NC_012920.1) and the human genome (GRCh38, chromosomes only) using CLC Genomics Workbench  9.0.1 “*Map Reads to Contigs”* analysis tool ([www.clcbio.com](http://www.clcbio.com)) at 95% and 99% alignment thresholds (Section B4).

**Figure S1. Agarose gel visualization of PCR products from conditions 2 and 3**

**
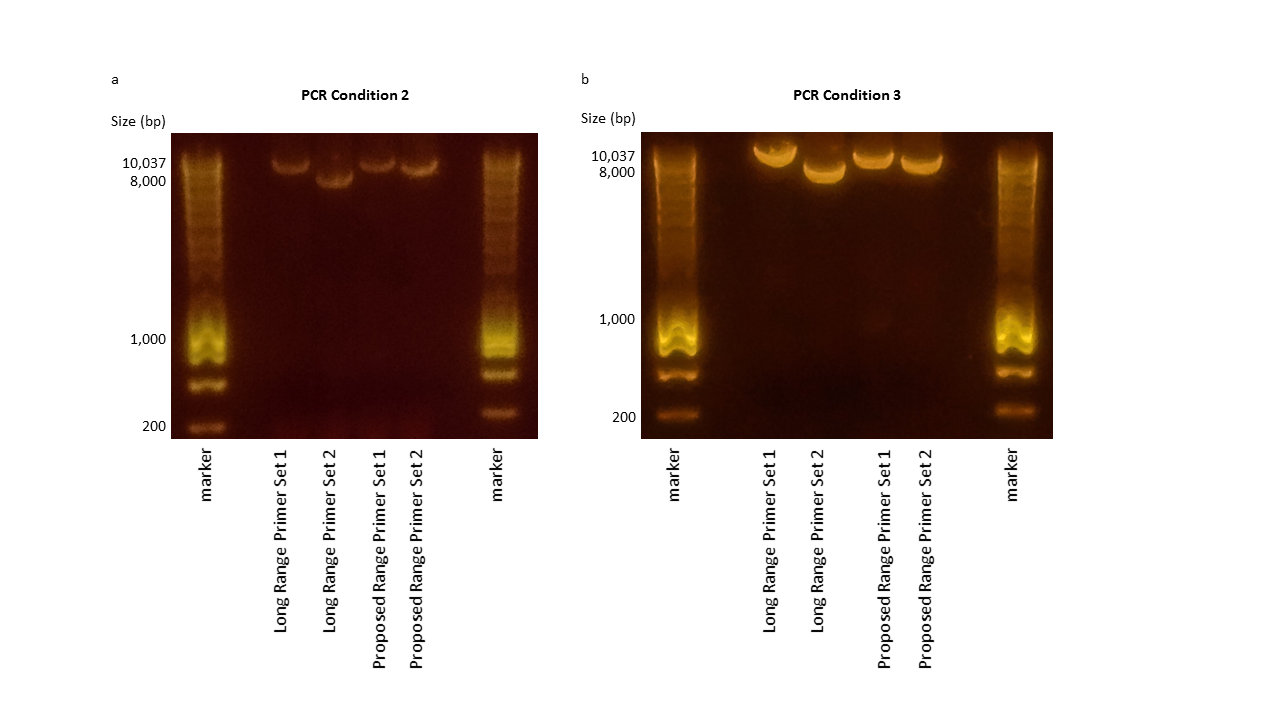
**

**Table S6. Proportion of mtDNA and nDNA reads in the sequencing data using 95% alignment threshold**

| Sample | Total Number of Reads | Reads Mapped to mtDNA only | Reads Mapped to mtDNA and nDNA | Reads Mapped to nDNA only | Unmapped Reads |
| --- | --- | --- | --- | --- | --- |
| Proposed PCR Primers Condition 1 | 1,739,656 | 824,145/47.37% | 885,050/50.88% | 6,978/0.40% | 23,483/1.35% |
| Long Range PCR primers Condition 1 | 3,173,090 | 1,329,433/41.90% | 1,799,600/56.71% | 10,025/0.32% | 34,032/1.07% |
| Proposed PCR Primers Condition 2 | 2,587,714 | 1,180,867/45.63% | 1,380,461/53.35% | 1,572/0.06% | 24,814/0.96% |
| Long Range PCR primers Condition 2 | 2,634,837 | 1,081,970/41.06% | 1,526,195/57.92% | 4,132/0.16% | 22,540/0.86% |

**Table S7. Proportion of mtDNA and nDNA reads in the sequencing data using 99% alignment threshold**

| Sample | Total Number of Reads | Reads Mapped to mtDNA only | Reads Mapped to mtDNA and nDNA | Reads Mapped to nDNA only | Unmapped Reads |
| --- | --- | --- | --- | --- | --- |
| Proposed PCR Primers Condition 1 | 1,739,656 | 1,179,611/67.81% | 156,405/8.99% | 8,106/0.47% | 395,534/22.74% |
| Long Range PCR primers Condition 1 | 3,173,090 | 2,173,975/68.51% | 372,482/11.74% | 14,867/0.47% | 611,766/19.28% |
| Proposed PCR Primers Condition 2 | 2,587,714 | 1,898,247/73.36% | 257,048/9.93% | 4,671/0.18% | 427,748/16.53% |
| Long Range PCR primers Condition 2 | 2,634,837 | 1,883,043/71.47% | 321,730/12.21% | 8,305/0.32% | 421,759/16.01% |

**References**

1. Lascaro D, Castellana S, Gasparre G, Romeo G, Saccone C, Attimonelli M: **The RHNumtS compilation: features and bioinformatics approaches to locate and quantify Human NumtS**. *BMC Genomics* 2008, **9**:267.

2. Simone D, Calabrese FM, Lang M, Gasparre G, Attimonelli M: **The reference human nuclear mitochondrial sequences compilation validated and implemented on the UCSC genome browser**. *BMC Genomics* 2011, **12**:517.

3. Qu W, Zhang C: **Selecting specific PCR primers with MFEprimer**. *Methods Mol Biol* 2015, **1275**:201-213.

4. Li M, Schonberg A, Schaefer M, Schroeder R, Nasidze I, Stoneking M: **Detecting heteroplasmy from high-throughput sequencing of complete human mitochondrial DNA genomes**. *Am J Hum Genet* 2010, **87**(2):237-249.
